# Polysaccharide Thin Solid Films for Analgesic Drug Delivery and Growth of Human Skin Cells

**DOI:** 10.3389/fchem.2019.00217

**Published:** 2019-04-09

**Authors:** Tina Maver, Tamilselvan Mohan, Lidija Gradišnik, Matjaž Finšgar, Karin Stana Kleinschek, Uroš Maver

**Affiliations:** ^1^Laboratory for Characterization and Processing of Polymers, Faculty of Mechanical Engineering, University of Maribor, Maribor, Slovenia; ^2^Department of Pharmacology, Faculty of Medicine, University of Maribor, Maribor, Slovenia; ^3^Faculty of Medicine, Institute of Biomedical Sciences, University of Maribor, Maribor, Slovenia; ^4^Faculty of Chemistry and Chemical Engineering, University of Maribor, Maribor, Slovenia; ^5^Institute for Chemistry and Technology of Materials, Graz University of Technology, Graz, Austria

**Keywords:** thin (blend) films, alginate, carboxymethyl cellulose, diclofenac, lidocaine, keratinocytes, skin fibroblasts

## Abstract

Chronic wounds not only lower the quality of patient's life significantly, but also present a huge financial burden for the healthcare systems around the world. Treatment of larger wounds often requires the use of more complex materials, which can ensure a successful renewal or replacement of damaged or destroyed tissues. Despite a range of advanced wound dressings that can facilitate wound healing, there are still no clinically used dressings for effective local pain management. Herein, alginate (ALG) and carboxymethyl cellulose (CMC), two of the most commonly used materials in the field of chronic wound care, and combination of ALG-CMC were used to create a model wound dressing system in the form of multi-layered thin solid films using the spin-assisted layer-by-layer (LBL) coating technique. The latter multi-layer system was used to incorporate and study the release kinetics of analgesic drugs such as diclofenac and lidocaine at physiological conditions. The wettability, morphology, physicochemical and surface properties of the coated films were evaluated using different surface sensitive analytical tools. The influence of *in situ* incorporated drug molecules on the surface properties (e.g., roughness) and on the proliferation of human skin cells (keratinocytes and skin fibroblasts) was further evaluated. The results obtained from this preliminary study should be considered as the basis for the development “real” wound dressing materials and for 3D bio-printing applications.

## Introduction

Thin films from polymeric materials present an emerging platform for various types of applications, including drug delivery (Maver et al., 2015b; Karki et al., 2016; Stana et al., 2017), sensors, microarray preparations (Mohan et al., 2012), etc. They also hold a huge scientific interest for several solid-liquid interface studies, especially for the adsorptive interaction of nanoparticles (Taajamaa et al., 2013), polyelectrolytes (Mohan et al., 2015, 2017), biomolecules (Kargl et al., 2015), etc. While significant efforts have been made to formulate drug delivery platforms from organo-soluble synthetic polymers (Vendra et al., 2011; Ponnusamy et al., 2012), the use of natural polymers (e.g., polysaccharides) for such applications is limited and have recently gained a paramount of interest (Zelikin, 2010; Park K. et al., 2018; Park S. et al., 2018). Due to their high compatibility, bio-inertness, diverse physicochemical properties and ease of handling during manufacturing, polysaccharide thin films are excellent candidates for skin-related applications, where a controlled and targeted delivery of pain-relieving analgesic drugs are prerequisite (Miao et al., 2018). Skin is the largest organ of the integumentary system and it plays a vital protective role of the human body against pathogens. Thus, any major injury (wound) that results in the loss of its integrity, can lead to significant patient disability or even death (Singer and Clark, 1999). Such injury/wounds are often further associated with severe pain that represents a stress for the patient and is therefore a potential factor in delaying the physiological process of wound healing (McGuire et al., 2006; Guo and Dipietro, 2010). Optimizing the analgesic treatment of injured patients is thus of major importance. In order to avoid several side effects that analgesic drugs have, the local application could be a good alternative (Palao i Domenech et al., 2008; Marques et al., 2014). However, there are difficulties in quantitively controlling and prolonging the release of analgesic drug molecules such as lidocaine (LID) and diclofenac (DCF), when they are employed without any supporting matrices. Several attempts have been made to incorporate LID and DCF into nanoscale multi-layered thin films for controlling their loading capacity and release profiles (Loo et al., 2010; Jeganathan et al., 2016). LID is among the most widely used local anesthetics due to its rapid action (the effect is noticed immediately), making it suitable to alleviate the pain, caused by dressing change (Zhao et al., 2018), while DCF is a long lasting non-steroidal anti-inflammatory drug, which can provide a longer-lasting pain reduction, caused by the injury itself (Altman et al., 2015).

Since LID and DCF are both water soluble and hydrophilic, their incorporation into thin films derived from hydrophobic polymers, are still challenging. Therefore, the polysaccharides [e.g., alginate (ALG) and carboxymethyl cellulose (CMC)], which are both inherently hydrophilic and water soluble, seem suitable candidates to incorporate large amounts of both mentioned drugs in a uniform fashion. ALG is the most commonly used mucoadhesive polymer in wound healing applications, owing to its excellent water uptake capacity, non-toxicity and abundance in nature (Pawar and Edgar, 2012). It is derived from brown algae and bacteria and is classified as a linear anionic polysaccharide, composed of repeating units of β-1,4-linked D-mannuronic acid (M) and L-guluronic acid (G) in varying ratios. Depending on the type of ALG, its composition leads to the formation of tight ionically cross-linked network structures; contributing to a prolonged drug release (Paques et al., 2014). CMC, a derivative of the most abundant natural polymer cellulose, is also anionic, weakly charged and water soluble. Both were already proven to be efficient film-forming polymers and have found use in erodible and other drug formulations (Palmer et al., 2011).

Until now, the layer-by-layer (LbL) self-assembly technique has been largely used to create nanoscale multi-layered systems, which are of interest for targeted drug delivery applications (Stana et al., 2017; Park K. et al., 2018), where the simplicity and versatility of the preparation procedure lower the overall drug delivery system costs. In general, the LbL technique exploits the alternating deposition of oppositely charged substances (e.g., polyelectrolytes, polypeptides, etc.), which enables surface and bulk material decoration with a wide range of functional groups, in the final multi-layer structure (Liu et al., 2019; Zhang et al., 2019). However, the application of this technique to create multi-layered nanofilms from a single polymer or mixture of polymers with the same ionic/functional groups, and the use of the latter system in the release of therapeutic drug molecules is still only scarcely reported (Finšgar et al., 2016; Stana et al., 2017). Amongst several other fabrication techniques, spin coating is the commonly used technique for the design of multi-layered structures. Its main advantages include a high reproducibility, reliability, rapid film formation, and simultaneous solvent evaporation. On top of the mentioned, it allows to control the thickness, morphology and surface properties of the final material (Stana et al., 2017). The challenges in the fabrication of multi-layered nanofilms from water soluble and hydrophilic polymers (with similar overall properties) such as ALG, CMC, and the ALG-CMC combination by spin-assisted LbL technique was not yet explored in a systematic way. Furthermore, the potential application of these self-assembled polysaccharide multi-layers for incorporation and controlled release of LID and DCF were also not yet investigated in detail.

The motivations for this work, therefore, was to create a nanoscale multi-layered thin films, as a model drug-carrier system, in a systematic way from hydrophilic ALG and CMC. In addition, this work also aims to gain a deeper understanding of the surface and physiochemical properties of the developed multilayered system. For this purpose, aqueous solutions of ALG, CMC, and the mixture of ALG-CMC with incorporated DCF or LID drugs were used to fabricate multi-layered thin films on different solid substrates by the spin-assisted LBL coating technique. The wettability, thickness, morphology, and roughness of the multi-layered films were characterized using several surface sensitive methods. The time-dependent release of drugs from the multi-layer at body temperature and at physiological pH were also determined. The obtained release profiles were further fitted with various kinetic models to discern the release mechanism of the drugs. Finally, the influence of the multi-layer films with *in situ* included drugs on the proliferation of human skin cells (keratinocytes and skin fibroblasts) was evaluated. The results obtained from this preliminary study should be considered as the basis for the development of new functional and active 3D materials to be used in regenerative medicine.

## Experimental Section

### Materials and Methods

All chemicals used for the preparation of multi-layered thin films (ALG: viscosity: 0.015–0.025 Pa s, CMC: Viscosity: 2.5–6 Pa s, DCF and LID) were purchased from Sigma-Aldrich, Germany. The tetrazolium salt MTT [3(4, 5 dimethylthiazolyl-2)-2, 5-diphenyltetrazolium bromide] for the biocompatibility testing was purchased from Sigma-Aldrich, Germany. Advanced Dulbecco's Modified Eagle's Medium (ADMEM) and Fetal Bovine Serum (FBS) were purchased from ThermoFisher, Germany. Keratinocytes, an aneuploid immortal keratinocyte cell line (HACAT) from adult human skin was kindly provided by Prof. Dr. Elsa Fabbretti (Centre for Biomedical Sciences and Engineering, University of Nova Gorica, Slovenia). Human derived skin fibroblasts (ATCC CCL-119, Detroit 551, LGC Standard) were purchased from ATCC, UK. For all experiments, ultra-pure water (18.2 MΩ cm at 25°C) was used, prepared from a ELGA Purelab water purification system (Veolia Water Technologies, UK).

### Sample Preparation

Three types of solutions were prepared for spin-coated. **Type I** (ALG-DCF or ALG-LID, 0.5/0.1%, w/v): 500 mg ALG was added to DCF or LID (0.1%, w/v, dissolved in water), stirred overnight with a mechanical stirrer. ALG (0.5%, w/v) was prepared in the same way without DCF or LID. **Type II**: (CMC-DCF or CMC-LID), CMC with and without DCF or LID with the same concentrations as mentioned in Type I were prepared. **Type III**. ALG-CMC-DCF or ALG-CMC-LID: ALG and CMC with DCF or LID were mixed together in a ratio of 1:1. The final concentration of the polymers and drugs in the final solution are 0.25 and 1 mg/mL, respectively. All solutions were prepared freshly before use.

### Multi-Layered Thin Film Preparation and Drug Incorporation

Silicon wafers were used as base-substrates for multi-layered thin film preparation. Prior to spin-coating, the silicon wafers were cut into pieces of 1 × 1 cm^2^, soaked into a “piranha solution” [H_2_O_2_ (30 wt. %)/H_2_SO_4_ (conc.): 1:7 (v/v)], rinsed with ultra-pure water and blow dried in a stream of dry nitrogen of high purity (99.999%). For creating multi-layers, the above-mentioned three types of polymer solutions with and without drugs were used. For spin-coated, 50 μL of ALG or CMC or ALG-CMC mixture with DCF or LID was deposited on the static substrate and spin-coated with a spinning speed of 4,000 revolutions per minute (rpm) and an acceleration of 2,500 rpm s^−1^ for 60 s. This procedure was repeated three times for each sample. In this way, three layers of the same material were created. These spin-coated final three layers from ALG, CMC, and ALG-CMC are designated as ALG 3, CMC 3, and ALG-CMC 3, respectively, without incorporated drugs. The drug incorporated and spin-coated three layers are designated as CMC-DCF 3 (or LID 3), ALG-DCF 3 (or LID 3), or ALG-CMC-DCF 3 (or LID 3).

### Atomic Force Microscope (AFM)

AFM was used for the topographical and surface roughness analysis of spin-coated multi-layered thin films. Prior to analysis using the Keysight 7500 AFM (Keysight Technologies, USA), the samples were attached onto round shaped metal disc sample holders. Topography images were acquired using the acoustic AC AFM mode. Silicon AFM tips (Nanosensors, Switzerland) with a nominal spring constant 12–110 N/m and a nominal resonance frequency of 210–490 kHz, were used for imaging purposes for all samples. Images of 1 × 1 μm^2^ were recorded, with a resolution of at least 524 × 524 pixels. All images were processed, and the corresponding roughness was calculated using freeware Gwyddion software package. For thickness determination, a straight line was created on the spin-coated sample by scratching with a sharp scalpel. Subsequently, a profile was created along the scratched straight line. This profile was then used to estimate the thickness of each spin-coated layer.

### Attenuated Total Reflection-Fourier Transform Infrared (ATR-FTIR) Analysis

The ATR-FTIR spectra of spin coated samples were measured using a Perkin Elmer FTIR System Spectrum GX Series-73565 at a wavenumber range of 4,000–650 cm^−1^. A total of 32 scans were performed for all measurements with a resolution of 4 cm^−1^. QCM quartz crystals (QSX301, LOT-Oriel, Germany) coated with a gold layer were used as substrates for ATR-FTIR measurements. The spin coating of polymer solutions with and without drugs were performed in the same way as with silicon wafers.

### Contact Angle Measurements

The wettability of spin-coated multi-layers was determined using a OCA15+ goniometer system (Dataphysics, Germany) with the sessile drop method. Static contact angle (SCA) measurements were carried out using ultra-pure water at ambient temperature. All measurements were carried out on at least two independent surfaces with a drop volume of 2 μL. Each SCA value was the average of at least five drops of liquid per surface.

### *In vitro* Release Studies

The release of DCF and LID from the multi-layered thin films was studied using an Automated Transdermal Diffusion Cells Sampling System (Logan System 912-6, Somerset, USA). For this purpose, each spin-coated wafer (1 × 1 cm^2^) was transferred to a 15 mL Franz diffusion Cell, filled with phosphate buffered saline (PBS, Sigma-Aldrich, Germany) with pH of 7.4. Its temperature was maintained at 37°C all the time. Samples were collected over a period of 24 h at different time intervals. The absorbances were determined using a UV-Vis spectrophotometer (Cary 60 UV/VIS, Agilent, Germany), and the released amounts calculated by quantification of the absorption bands at 276 nm (DCF) and 204 nm (LID), using the Beer-Lambert law. The withdrawn sample volumes were replaced by fresh PBS. During the release profile calculations, this dilution was accounted for. The release measurements of at least three parallels of each sample were conducted and the results are reported as average value with ± standard error.

To evaluate the release profiles further, the results from the *in vitro* drug release test were fitted using three known models (first-order, Higuchi-model, and Korsmeyer-Peppas model). Using these models (Costa and Sousa Lobo, 2001), the release mechanisms of the respective drugs from the multi-layers were analyzed.

First-order kinetics (1) is used to describe the release of the drug where the release rate is concentration depended.

(1) $$\begin{array}{r} \log\text{Q}=\log{\text{Q}}_{0}-{\text{K}}_{\text{t}}/2.303 \end{array}$$

where Q is the amount of released drug in time “*t*,” Q_0_ is the initial concentration of the drug and *K* is the first-order rate constant.

Higuchi-model (2) is used to express the drug release from an insoluble matrix as a square root of time dependent process based on the Fick's diffusion:

(2) $$\begin{array}{r} \text{Q}=\text{K}{\text{t}}^{1/2} \end{array}$$

where *Q* is the amount of drug released at time “t” and K is the Higuchi constant.

The Korsmeyer-Peppas model (3) describes the release of drugs from polymeric systems, which has been successfully applied to describe the release from different modified release dosage forms:

(3) $$\begin{array}{r} {\text{Q}}_{\text{t}}/{\text{Q}}_{\text{∞}}={\text{Kt}}^{\text{n}} \end{array}$$

where Q_t_ is the amount of drug released in time “t,” Q_∞_ is the amount of drug released after infinite time, K is the constant, and n is the diffusional exponent. If the n value is equal 0.5, then the release mechanism is Fickian diffusion, if 0.45 < *n* < 0.89, then it is called non-Fickian or anomalous diffusion. In the case of “*n*” is between 0.89 and 1, and above 1, the drug release is the combination of both diffusion and erosion-controlled processes.

### Biocompatibility Testing

In order to test the influence of added DCF and LID on cell viability, the latter was evaluated via the reduction reaction of the tetrazolium salt MTT [3(4,5 dimethylthiazolyl-2)-2,5-diphenyltetrazolium bromide], purchased from Sigma Aldrich, Germany. This is a commonly used and reliable method to evaluate cell proliferation (Finšgar et al., 2016; Maver et al., 2018). It is based on the reduction of the yellow MTT by metabolically active cells (e.g., in part by dehydrogenase enzymes), which results in the formation of nicotinamide adenine dinucleotide and/or nicotinamide adenine dinucleotide phosphate (Maver et al., 2017). During this reaction, a purple formazan is formed intracellularly, which can be solubilized and quantified by spectrophotometric means. Thin films on silicon wafers (1 × 1 cm^2^) were exposed to the UV light for 30 min (i.e., sterilization). Further, the samples were soaked into 1.5 mL of Advanced Dulbecco's Modified Eagle's Medium (ADMEM; ThermoFisher, Germany), supplemented with 5 wt.% Fetal Bovine Serum (FBS; ThermoFisher, Germany), and incubated for 24 h at 37°C in an atmosphere containing 5 wt.% CO_2_. The HaCaT and Skin Fibroblast cells (10,000 cells/well) were seeded into a 96-well microtiter plate with a final volume of 100 μL of ADMEM medium supplemented with 5 wt.% FBS. The material samples (supernatants of the starting samples) were added to the cells after 24 h of incubation at 37°C in an atmosphere containing 5 wt.% CO_2_ in four parallels. As control, ADMEM medium supplemented with 5 wt.% FBS was added to the cells. After 24 h of treatment, cell viability was determined using the standard reduction of the tetrazolium salt MTT (Stergar et al., 2017; Maver et al., 2018b).

### Statistical Analysis

All numerical values are given as average values ± standard deviation. Statistical analysis was performed using SPSS Statistics 25 (IBM Corp. Armonk, NY, USA). By using the Shapiro–Wilk test it was determined that all experimental data was normally distributed, allowing the use of one-way ANOVA. *P* < 0.05 were considered statistically significant (these are marked with an asterisk ^*^ in respective graphs).

## Results and Discussion

### Surface Morphology, Layers Thickness, and Wettability

#### Surface Morphology

**Figure 1:**
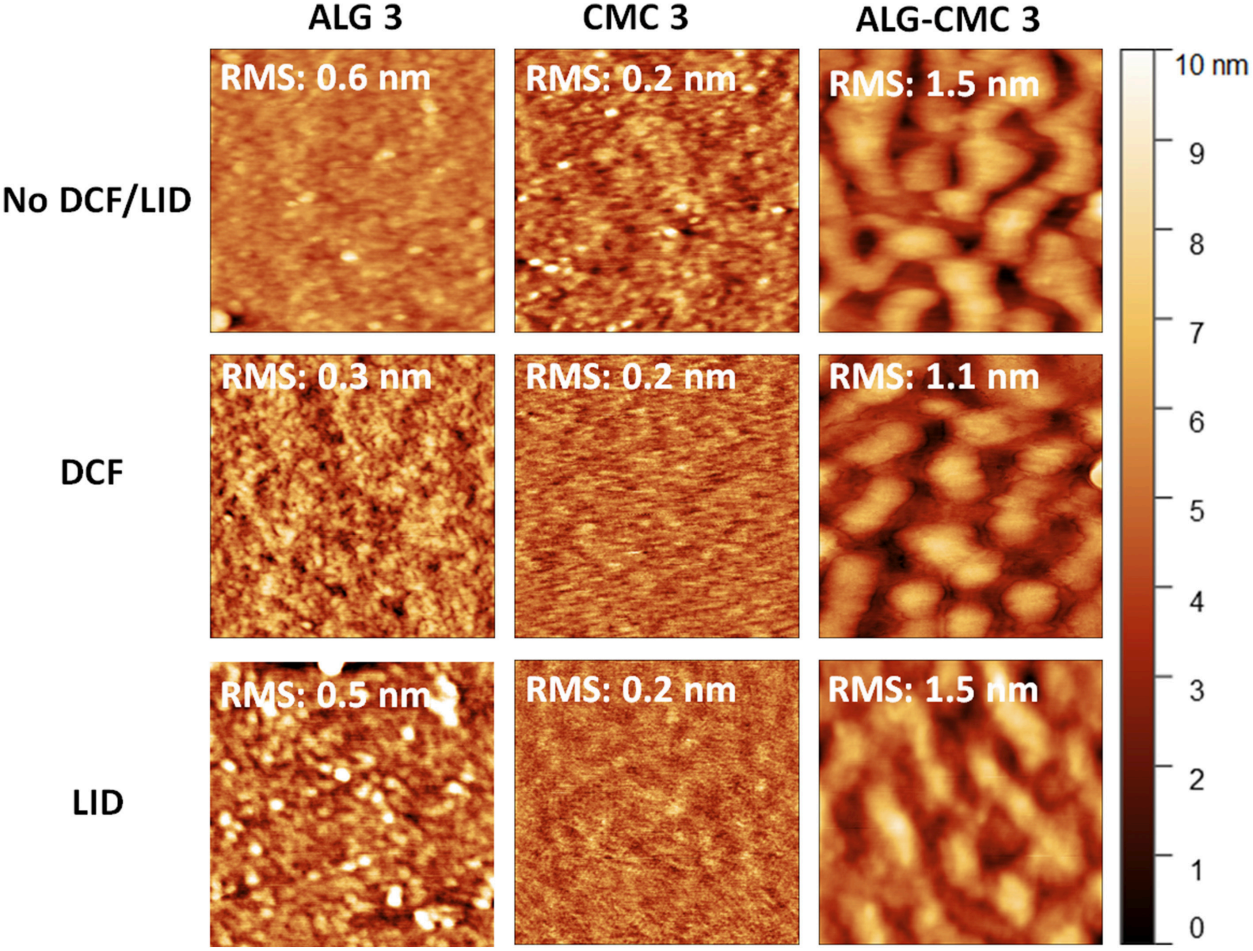
AFM height images (size: 1 × 1 μm^2^) of polysaccharide nanofilms, without drugs **(top)**, with DCF **(middle)**, and with LID **(bottom)**.

**Figure 2:**
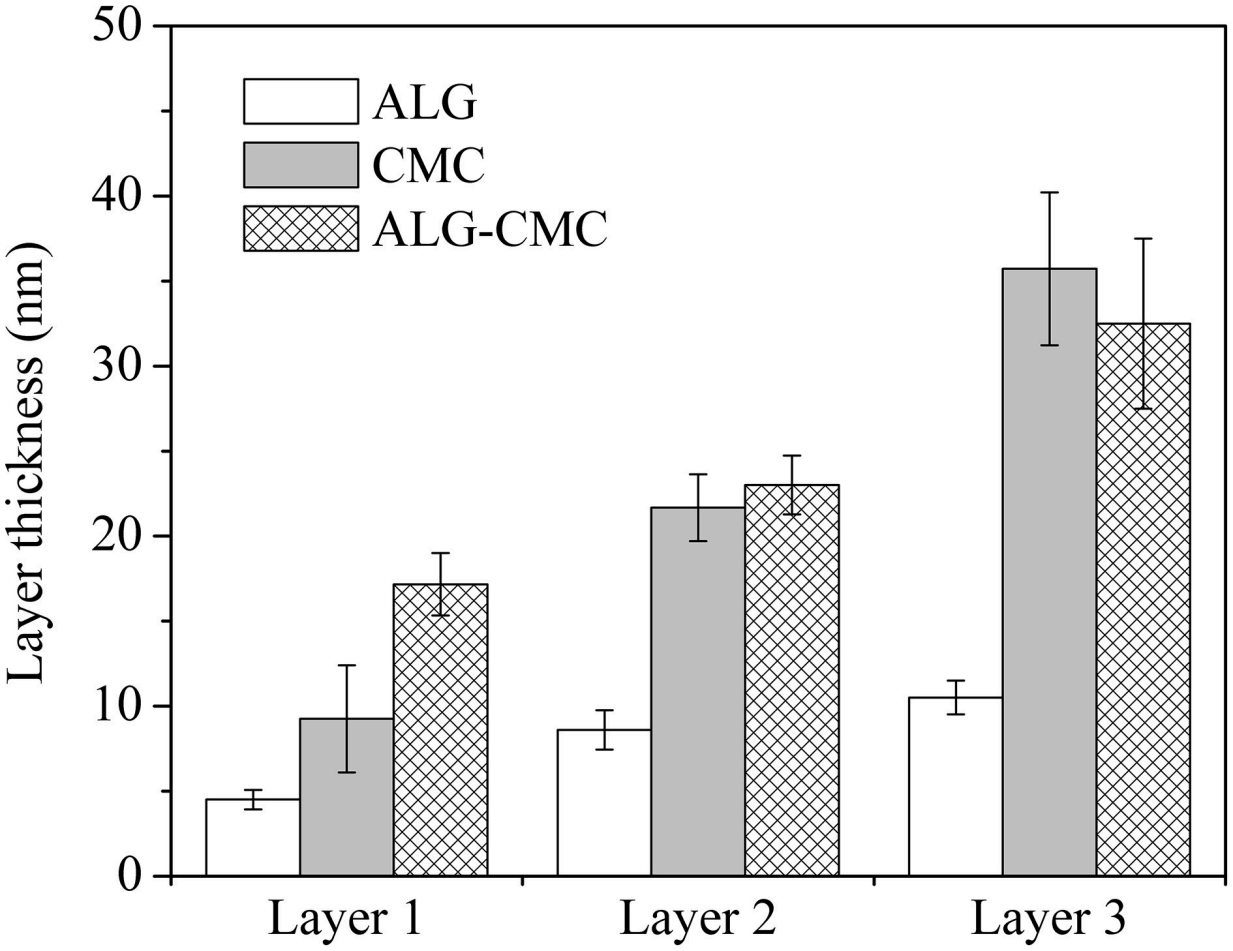
Layer thickness of spin-coated films determined using AFM.

The surface morphology and roughness of the spin-coated multi-layer films based on ALG, CMC, and the ALG-CMC mixture were characterized using atomic force microscopy (AFM). For clarity reasons, only the results obtained from the final (top) layers are shown in this section, since the same system is used for the release of DCF and LID drugs (section *In vitro* Release Studies). While smooth morphologies with a low surface roughness value (below 1 nm) are observed for both drug-free ALG 3 and CMC 3 coated films, the ALG-CMC mixture prepared at 1:1 ratio (top right image in Figure 1) did show some different surface features. Interestingly, the surface roughness of both ALG and CMC 3 samples is lower when compared to its layer thickness (see Figure 2). This is most likely due to tight packing and more self-organization of polymer molecules within the film structure. Surfaces with a phase separated morphology are detected for the two-component system that are composed of same charges i.e., ALG-CMC 3. Such phase separation is already noticed for the first ALG-CMC layer (Figure S1).

The reports on the phase separation of polymer blends from (organo-soluble) hydrophobic polymers upon spin-coating are manifold (Taajamaa et al., 2011; Strasser et al., 2016). Whereas, no studies on the formation of blend surfaces from spin-coated polysaccharides, which are hydrophilic and water-soluble, can be found to the best of our knowledge. Therefore, maximum efforts are made to explain the observed lateral phase separation in the ALG-CMC 3 multi-layered system and not to overinterpret the results at the same time. At low solute (polymer) concentration, the polymer-polymer blend in solution stays in one phase only (Doublier et al., 2000; Keating, 2012; van de Velde et al., 2015). Upon spin-coating, the increase of solute concentration will push the system into two phases with each phase enhanced in its respective component. The formation of two phases (i.e., phase separation) is often a complex phenomenon, leading to the formation of non-equilibrium morphologies, due to the rapid removal of solvent during the spin-coating process. Usually, this leads to different structures/morphology on the nano- to micro-meter scale (Walheim et al., 1997). This nano- and micro-phase separation can be classified into associate and segregate, which in turn dependent on the affinity between polymers and solvent. The associate phase separation arises when both polymers are oppositely charged, and both polymers are intensified in one of the separating phases while leaving the other phases mostly with solvent. In the case of segregate phase separation, two polymers with same charges are separated into two different phases (Fang et al., 2006). The segregate phase separation is well-known for water soluble polymers (e.g., proteins- polysaccharides) for several years (Doublier et al., 2000), but not reported for spin-coated blend films. Keeping this mind, we believe that the lateral phase separation that occurred in the case of ALG-CMC blend, which stays in one phase in water, is of the segregate type, which can be explained by the transient bilayer theory (Heriot and Jones, 2005; Taajamaa et al., 2011).

According to the transient bilayer theory, the formation of the lateral phase separated structures/morphologies during spin-coating, occurs because of de-wetting and interfacial instabilities between the two polymers (ALG and CMC in this study). The instability at the interface is presumably caused by the solvent-concentration gradient within the film. Usually, the evaporation of the solvent is faster at the film surface than in the bulk phase, leading to a lower solvent concentration at the surface than at the polymer-substrate interface. Thus, the instability, caused by the interfacial tension at a polymer-polymer interface is a function of solvent concentration. Concretely this means that if one of the polymers out of the two polymer blend exhibits a strong interaction with the substrate, the phase separation is initiated in a surface-oriented fashion, resulting in a transient bilayer (Geoghegan et al., 1994; Walheim et al., 1997). With the evaporation of more solvent from the film, the upper layer of the film gets unstable with the formation of holes. The latter is then filled with the lower (liquid phase) layer as it is shown for ALG-CMC coated (first) layer (Figure S1) (Taajamaa et al., 2011). Interestingly, the phase separation is retained even after the third layer (Figure 1, top row and Figure S2). Using the phase image (Figure S2), the existence of two phases, with a thicker region surrounded by a thinner one, from the two-component system can be easily seen. However, we are aware of the fact that it is difficult to confirm the thicker or major phase is either ALG or CMC; which needs further investigation. From the above discussion, it is proposed that the lateral phase separation occurs upon spin-coating for the hydrophilic polysaccharides that carry similarly charged chains. The detailed understanding of these phenomenon at different ALG-CMC ratios (in water) and humidity' s is currently in progress and is out of the scope of this work. The addition of drugs (LID and DCF) leads to a change in surface morphology for both ALG 3 and CMC 3 coated layers (but not for the ALG-CMC 3 mixture). Especially, particle-like features are visible for LID incorporated ALG 3 sample, whereas a featureless and even more smooth morphology is noticed for both DCF and LID incorporated CMC 3 sample. This can be due, on one hand, an enhanced interaction between LID and functional groups in the multilayer system (as confirmed by IR) and on the other hand, potentially significant inter-diffusion of small, hydrophilic LID molecules within the entire film architecture during the film formation, which occurs during the spin-coating process. In the case of drug incorporated ALG-CMC 3 blend, the phase-separation is still visible with only minimal changes in the morphology. This could be due to the enrichment of drugs in both phases, as confirmed by ATR-FTIR and *in vitro* release studies, where the peaks correspond to DCF and LID and a successful release of both drugs is observed (see below).

#### Layer Thickness

The thicknesses of each spin-coated layer (for respective samples) are shown in Figure 2. Expectedly, the layer thickness increased with the number of layers deposited for all spin-coated samples. However, there is a difference among the spin-coated materials. For ALG based samples, a layer thickness of only ~10 nm is measured even after the spin-coated of the third layer. Whereas, a value close to 10 nm is already obtained for the first layer in CMC based samples. With the second and the third layers, the thickness value for this sample is increased from ~22 to ~35 nm, respectively. This can be (at least partially) explained by a higher molecular weight (700 kDa) and viscosity (2.5–6 Pa s) of the CMC solution, leading to deposition of higher polymer masses on the surface upon spin-coating. As can be seen in Figure 2, the thickness of the first layer of ALG-CMC blend is considerably higher when compared to the first layer of single component ALG and CMC (ALG-CMC: 17 nm > CMC: 9 nm > ALG: 5 nm). These results demonstrate that a multi-layer can be fabricated from a single or mixture of polysaccharides with the same functional groups (i.e., –COOH). In addition, it has to be pointed out that the layer thickness of drug-incorporated spin-coated films (of ALG, CMC or ALG-CMC blend) did not increase considerably compared to films prepared without drugs (data not shown). This could be due to lower concentration (see Figure 5) and homogenous incorporation of the drugs into the spin-coated layer. This gained knowledge, in future, can exclude the use of oppositely charged components, such as carboxymethyl cellulose—chitosan (Mohan et al., 2015), hyaluronic acid- chitosan (Pérez-Álvarez et al., 2019), alginate-chitosan (Stana et al., 2017; Criado-Gonzalez et al., 2019), etc., to create multi-layered system.

#### Wettability

The contact angle values directly reflect surface wettability and are sensitive to chemical functionalities of the outermost layer (Stana et al., 2017). The surface wettability of spin-coated multi-layers, incorporated with and without drugs, were measured by the static water contact angle [SCA(H_2_O)] measurements and the results are depicted in Table 1 (also see in Table S1). It shows that the spin-coated layers of ALG 3, CMC 3 or their combination are hydrophilic [obtained SCA(H_2_O) values < < 90°] (Yuan and Lee, 2013). For all three spin-coated samples, higher SCA(H_2_O) values are observed compared to the SCA(H_2_O) values of the substrate used for spin-coated [Si-wafers; SiO_2_ surface (10 ± 0.2°)] (Stana et al., 2017). The changes in the SCA(H_2_O) indirectly also confirm a successful deposition of the spin-coated polysaccharide materials. Compared to ALG 3 alone, slightly increased SCA(H_2_O) values for CMC 3 and the ALG-CMC 3 blend are observed, which can be related to the rougher and thicker surfaces of the latter samples (Figures 1, 2). Another important observation based on these results is that no further (major) changes in the SCA(H_2_O) values resulted for the incorporation of either of the drugs. Namely, hydrophilic and highly wettable surfaces are already on their own beneficial for wound healing of specific (e.g., chronic) wounds and changes toward more hydrophobic values would potentially limit their therapeutic efficiency. Furthermore, such surfaces can even enhance the drug release efficiency by allowing penetration of body fluids and thus enabling the dissolution and/or diffusion of the drugs from the materials to the wound area (Maver et al., 2018a).

**Table 1:** SCA(H_2_O) values of ALG, CMC, and ALG-CMC spin-coated multi-layers, incorporated with and without drugs.

	**ALG 3 [°]**	**CMC 3 [°]**	**ALG-CMC 3 [°]**
No drug	21 ± 1	29 ± 3	30 ± 2
DCF	23 ± 2	36 ± 1	33 ± 2
LID	24 ± 2	34 ± 3	31 ± 1

#### Surface Composition

**Figure 3:**
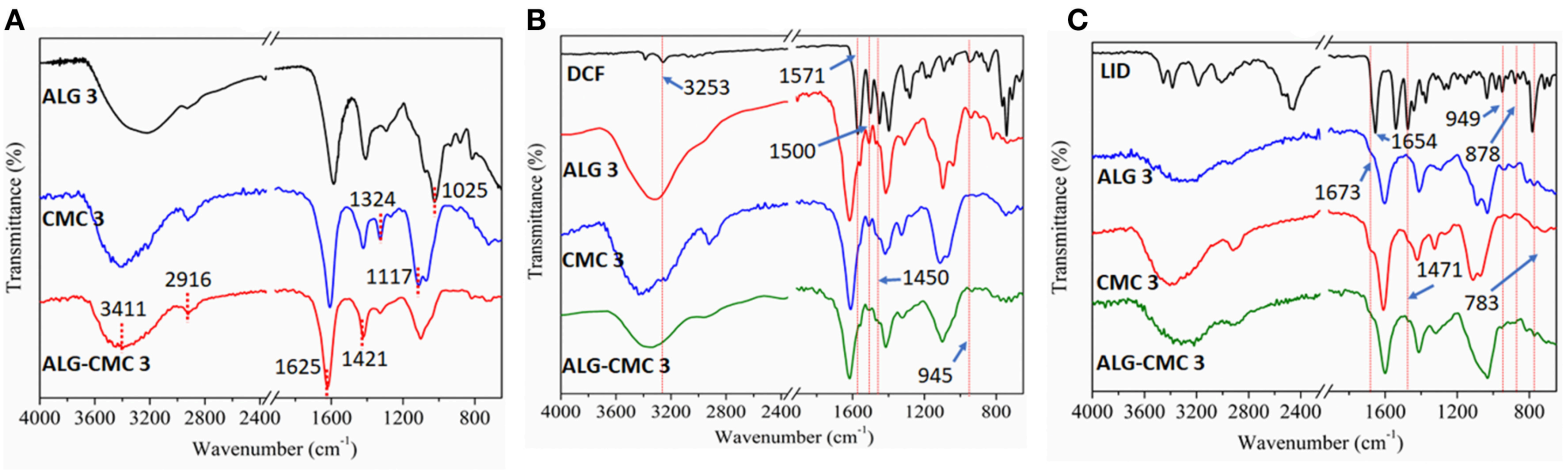
ATR-FTIR spectra of drug-free **(A)**, DCF-loaded **(B)**, and LID-loaded **(C)** polymers.

The surface composition of spin-coated multi-layers incorporated with and without drugs was analyzed using ATR-FTIR spectroscopy. Figure 3A shows characteristic peaks of pure ALG and CMC. For ALG, the observed peaks at 3,360, 2,903, and 1,625 cm^−1^ are attributed to O-H stretching, -CH stretching, the asymmetric and symmetric vibrations of -COO and C-O-C stretching, respectively. Whereas, all major peaks of CMC are observed at 3,411, 2,916, 1,583, 1,420, and 1,324 cm^−1^, which are assigned to O-H and –CH stretching, and the asymmetrical and symmetrical vibrations of carboxyl groups in the CMC, respectively. Although no additional new peaks are noticed for ALG-CMC 3 coated layers, all the characteristic peaks, as shown above, of ALG and CMC are detected. These results suggest that the ALG-CMC 3 layer is composed fully of both polymers and no new bindings/interactions are involved between the functional groups.

The spectra of sodium salt of DCF and DCF loaded polymers are shown in Figure 3B. Characteristic DCF peaks include at 3,253 cm^−1^ the NH stretching of the secondary amine, at 1,571 cm^−1^ the –C = O stretching of the carboxyl ion, at 1,500 cm^−1^ the C = C ring stretching, at 1,450 cm^−1^ the -CH_2_ bending, at 945 cm^−1^ the -C-O-C stretching, and at 746 cm^−1^ the C-Cl stretching. All these peaks are also present for all samples (ALG, CMC, and the ALG-CMC blends). Figure 3C shows the characteristic peaks of LID at 1,673 cm^−1^ for the stretching of carbonyl group, and at 1,541 cm^−1^ the in-plane bending of the N-H group. Also, in the case of the LID, most of these peaks are detected for all the polysaccharide based multi-layer samples. Interestingly, the -C = O peak position of CMC and ALG is altered after the incorporation of both drugs. The -C = O peak is shifted from 1,625 to 1,604 cm^−1^ and from 1,583 to 1,613 cm^−1^ for ALG and CMC, respectively. These peak shifts indicate that both drugs are involved in some sort of interactions, most likely ionic or H-bonding. Such a scenario is mostly favorable and desired for applications, especially for prolonging the release rate of the drugs. In addition, the drug molecules could also be physically incorporated, which has other advantageous that drug molecules could simply diffuse through the layers and be released into the medium or at the wound site without any hinderance from the polysaccharide in which the drugs are incorporated. It can be concluded that the ATR-FTIR results confirmed the presence, and hence a successful incorporation of both used drugs into the formed multi-layer systems regardless of their compositions. Therefore, the next step was a thorough drug release analysis of all drug incorporated samples.

### *In vitro* Release Studies

Controlled and local release of pain relieving, and anti-inflammatory drugs presents an important step toward a more successful chronic wound healing. Such drugs can not only diminish the pain from the wound itself, but also reduce the pain induced stress, which is known to hinder the healing efficiency, especially in the long term (Maver et al., 2015a). The used drugs herein (LID and DCF), come from different pharmacodynamic groups (LID is a local anesthetic and DCF a non-steroidal anti-inflammatory drug), and hence lower the sensation of pain through different mechanisms, improving the overall therapeutic potential of the developed multi-layered materials even further (Maver et al., 2017). Since they are both water soluble, DCF and LID can be easily mixed and homogeneously incorporated within the herein used hydrophilic host materials (ALG and CMC). Altogether, such systems provide an excellent platform for rapid design of novel multifunctional wound dressing materials as was shown before (Maver et al., 2019). In this study, novel polysaccharide drug loaded multi-layer thin film systems were fabricated that serve as a representative model of “real” dressing materials, enabling their optimization already during development. Since it is important to ensure an efficient pharmacotherapy i.e., controlled drug delivery for the newly developed systems, a lot of emphasis has been put on the evaluation of the results obtained from the *in vitro* drug release studies.

**Figure 4:**
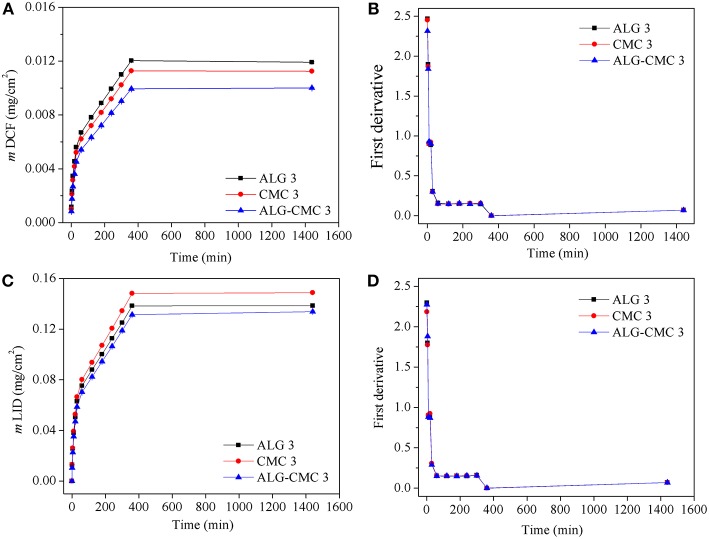
*In vitro* release studies of DCF and LID from the spin-coated multi-layers of alginate (ALG 3), carboxymethyl cellulose (CMC 3) and ALG-CMC 3 blend. Dissolution profiles of DCF **(A)** and LID **(C)** of mass change as a function of time. First derivative of the release data of DCF **(B)** and LID **(D)**.

The DCF and LID release profiles from the different polysaccharide multi-layers are shown in Figure 4. The release of both drugs is performed at physiological pH with PBS buffer and at a simulated body temperature (37°C) using the “Franz-diffusion” cell-based drug release system, which is the best currently available system to simulate the release from wound dressings. The released mass of DCF as a function of time is depicted in Figure 4A. Immediately it can be observed that there are notable differences in the released mass of DCF from the three systems. As it seems, the DCF release is favored from the “pure” ALG multi-layers, which is not really surprising considering that DCF is in its essence an “acid.” Whereas, CMC can exhibit multiple carboxylic groups per one sugar unit, this is not the case with ALG, which has only one such group in its structure. Therefore, increased repulsive forces could be present in the case of CMC multi-layer systems, which probably led already in the preparation steps to smaller incorporated DCF amounts, hence finally contributed to a smaller overall DCF release. In addition, as can be seen from the calculated first derivatives of the release data (Figure 4B), the profiles of all systems show that multiple mechanisms might be involved in the release of DCF as the time progresses (breaks in the curves of first derivatives). This corresponds to the fact that release of hydrophilic drugs from hydrophilic multi-layer thin films present not just a “simple” diffusion-controlled mechanism but is often further accompanied by a combination of swelling and erosion of polymer chains upon contact with a buffer solution. As soon as the drug becomes soluble and polymer chains becomes mobile, the diffusion of drugs through the multi-layered films occurs with a different rate. A careful evaluation of the release profiles shows a fast (burst) release (~45%) within the first 30 min, which most likely corresponds to the release and dissolution of “trapped” or unbound drug molecules within the polymer structure. The latter is followed by a slower and prolonged release (~50%) for the next 6 h (360 min), which is related to the combination of swelling and partial erosion (mentioned above). The last release stage is the so called “plateau,” which present a diffusion-controlled release of the remainder of the drug from the remaining (mostly intact) part of the multi-layer system. Another important observation based on the obtained release results is that regardless of the composition, all three different samples seems to follow the same combined release mechanism (confirmed by the calculation of the first derivatives shown in Figure 4B). This has another implication for the potential clinical use of such materials. Namely, by simply choosing another polymer (or the combination thereof), the physician can also control the overall used/delivered drug dose to the wound. Further studies would be necessary to show how we can further control the released drug amount by changing the incorporated drug amount, but this is out of the scope of this study. Developing the thought about the potential clinical application of such materials further, it can be said that such a release profile is essentially as desired in release of analgesic drugs in chronic wound treatment. Whereas, the initial “large” released amount of the analgesic drug is essential to provide a strong pain-relieving action as soon as possible, the sustained release is important to lower the pain sensation until the dressing is changed. The sustained drug release further improves the quality of life of the patient in long-term during treatment.

**Figure 5:**
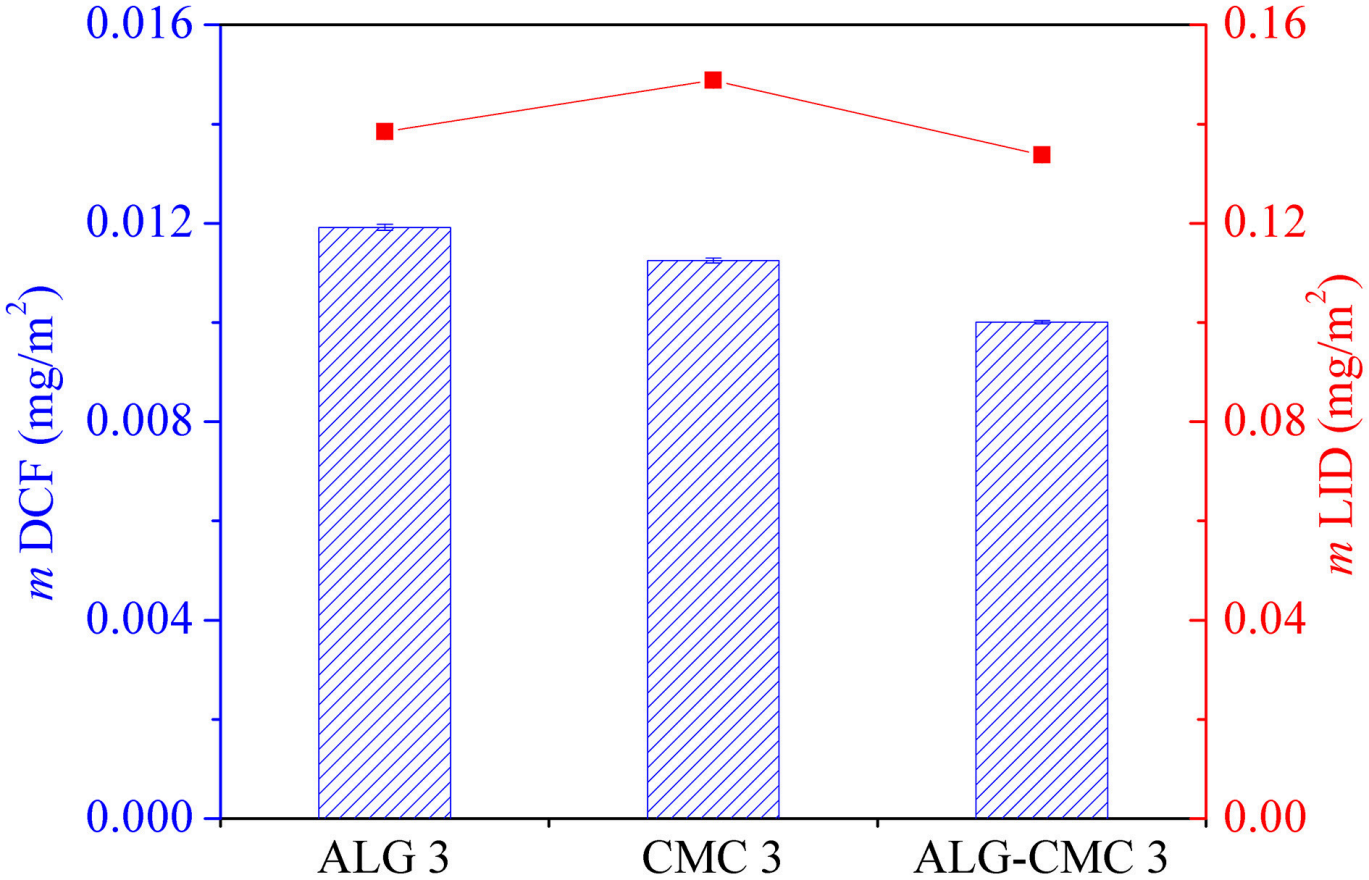
Total amount of released drug mass from different spin-coated polysaccharide multi-layered thin films.

Similar deductions about the release mechanism can be made for the system with incorporated drug LID, although there is a different order of the systems in regard of the total release LID amount. Namely, here the CMC based system show the highest release amount, which is most likely again connected with the polymer and drug molecular structure. LID presents an amide, which has another secondary amine in its structure. The latter is capable to form interactions (e.g., ionic bonds) with carboxylic groups, which might have contributed to a higher incorporated dose of the drug during the preparation step (i.e., spin-coating). Since CMC has a higher amount of such groups, more of the drug could have been interacting with the polymer chains in the multi-layer film structure. This data fits with the ATR-IR results where the shift of -C = O peaks of polymers are observed as the result of ionic interactions. Two more conclusions can be made based on the results shown in Figure 4. Firstly, the multi-layer systems with incorporated LID had a higher overall incorporated amount of the drug. And secondly, regardless of the drug type, the combined polymer system (ALG-CMC) has shown the most sustained release. To ease further discussions of the latter, the overall released mass of DCF and LID from three types of multi-layers are compared and the results are shown in Figure 5. The total released mass of DCF is higher for ALG 3 with a thickness of *ca*.11 nm than the other two multi-layers (CMC 3: 36 nm, ALG-CMC 3: 33 nm). It is suggested that the DCF as a small molecule with a molecular weight of 296.2 g/mol is expected to diffuse faster in and out of the thin multi-layer films. From this release result, it can be stated that even the thinnest multi-layer films can be used to incorporate and release drugs in therapeutic doses in a controlled manner similar to that of three times thicker CMC 3 or ALG-CMC 3 films. Among other two multi-layers, a slightly lower mass is found for ALG-CMC 3. A reason can be that the diffusion of DCF through the thicker ALG-CMC 3 and hierarchically structured film from the two polymer components is prevented, and thus the overall released mass is lower. However, a different trend is observed for LID incorporated multi-layers. While ALG 3 and ALG-CMC 3 showed almost the same amount of the released mass, CMC 3 multi-layers showed a slightly higher mass release, which is the opposite to the results observed for DCF incorporated CMC 3 multi-layer. For LID incorporated layers (Figure 4C), although the release profile is similar to DCF, the overall released mass is 10 times higher when compared to DCF. This can be related to differences in the solubility of both drugs. It is suggested that upon contact with water, compared to DCF (solubility in water: 0.005 mg/mL), the hydrochloride form of LID (used in this study, solubility in water: 0.6 mg/mL) incorporated within the hydrophilic ALG-CMC 3 matrix dissolves and diffuses quicker through the film, and thus a larger amount of drug is released over time. Overall, the data show that it is possible to carefully tune the release rate of drug using one particular system, as it is demonstrated by several others (Vasilev et al., 2011; Wang et al., 2016; Maver et al., 2018a; Speer et al., 2019). Using thinner and thicker multi-layer coatings, the release rate can be extended even further (desired in treatment of chronic wounds is for multiple days).

**Figure 6:**
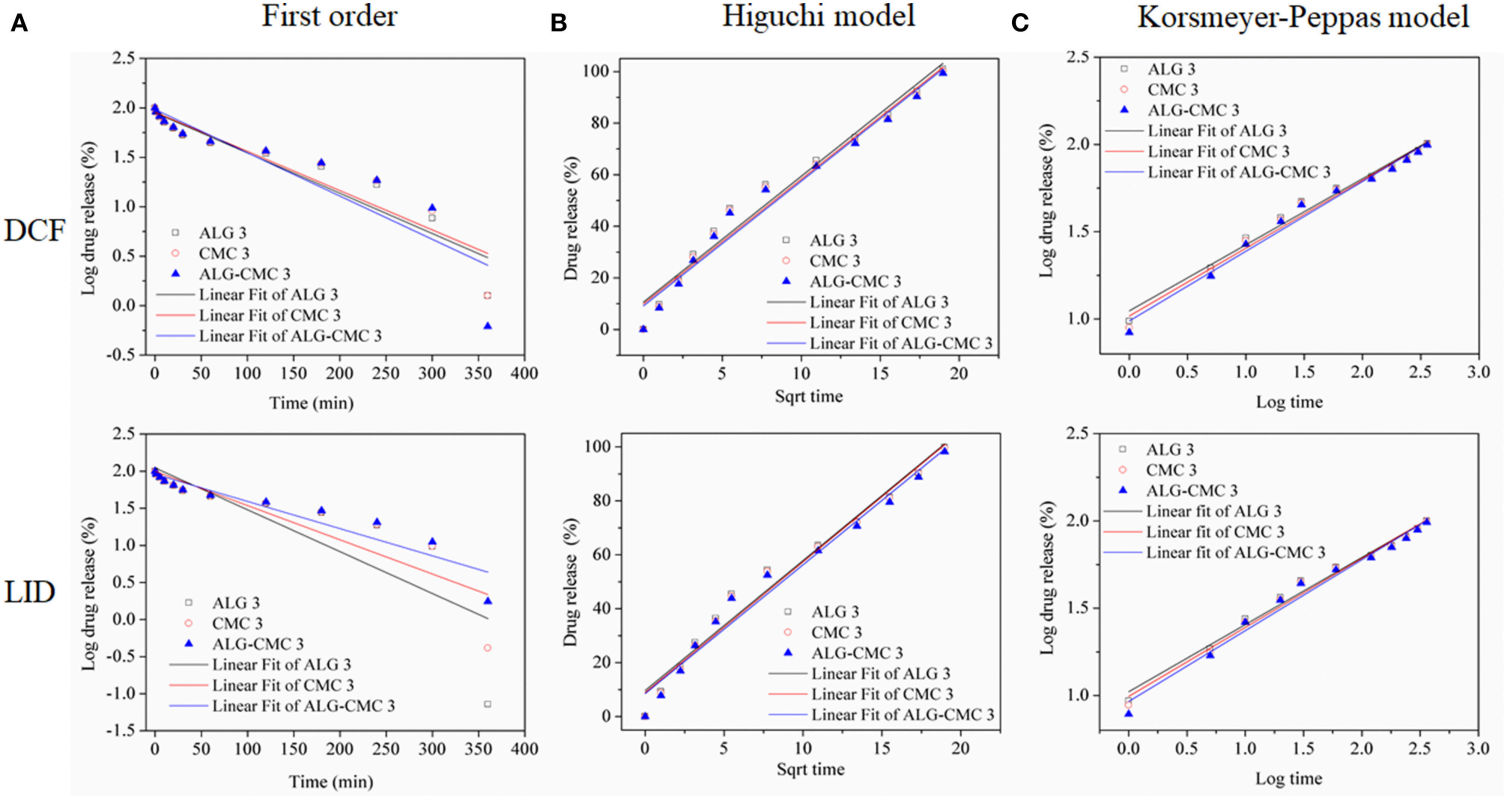
Drug release data of DCF and LID loaded polymers fitted to various kinetic models.

To further evaluate the kinetics and mechanisms of drug (DIC and LID) release from the multi-layers, the *in vitro* release data were fitted by linear regression analysis according to different kinetic models. Although different kinetic models were considered, only the models (First-order, Higuchi-model, and Korsmeyer-Peppas model, see Table 2) that gave reasonable fits for the release data were selected [based on the square of the correlation coefficient (*r*^2^) value]. The kinetic model that resulted in the highest *r*^2^ value was considered as the best fit for the release data. Among the tested three kinetic models, the Korsmeyer–Peppas model had the best fit with the highest *r*^2^ value for all multi-layers. Slightly lower *r*^2^ values were calculated for the Higuchi-model (reflected in Table 2 and Figure 6). Since the first-order model resulted in much lower *r*^2^ values, it was not considered for further interpretation and discussion of the results. Considering the best fit (highest *r*^2^ value in Table 2), as well as considering the calculated first derivative results based on the release data (shown in Figures 4B,D), it can be assumed that more than one mechanism is involved in the release of the drug from all three multi-layers (Keny et al., 2009). For the fitting of the results, the release data from 0 to 360 min were used. The best fit was obtained with Korsmeyer-Peppas model, which is among the most common models to describe the drug release kinetics from polymeric systems, when the release mechanism is unknown, or more than one release phenomena is involved. The release component “*n*” value from the Korsmeyer–Peppas Equation (3) was shown to be related (at least partially) to different release mechanisms and can therefore be used for an estimation of the latter. From Table 2 it can be seen that the “*n*” value is found to vary with type of multi-layers used, whereas the absolute value is in all cases above 0.89 for both drugs. This calculation leads to the conclusion that the release of both drugs from all system is an anomalous non-Fickian diffusion transport (super case-II transport mechanism). This means that the drug molecules are released through a diffusion process in a highly hydrated polysaccharide matrix that involved in the dissolution or relaxation of polymer chains (might be either swelling, erosion, or both) (Nayak and Pal, 2013). Since the Higuchi model came close to the best fit (*r*^2^: 0.96–0.97), let us consider what it would mean, if the latter model would hold. Considering that the Higuchi model presents the derivation of an equation that allowed for the quantification of drug release from thin ointment films, containing finely dispersed drug (applicable in perfect sink conditions), this model could also potentially be used to explain the release mechanism in this study. For example, the obtained *r*^2^ values suggest that the release of drugs from the multi-layered system is controlled by diffusion, which is totally opposite to non-Fickian diffusion approach. Therefore, according to the best fit and highest *r*^2^ value, we believe that the release of both drugs from all three multi-layered system follows Korsmeyer–Peppas model and no other kinetic models.

**Table 2:** The values of correlation coefficient (*r*^2^) and release exponent (n) calculated from different kinetic models for DCF and LID loaded system.

**Samples**	**First order**	**Higuchi model**	**Korsmayer-Peppas model**	
	***r*^**2**^**	***r*^**2**^**	***r*^**2**^**	***n***
ALG 3	0.900	0.966	0.984	1.05
CMC 3	0.878	0.966	0.981	1.02
ALG-CMC 3	0.816	0.969	0.981	0.99
ALG 3	0.683	0.969	0.985	1.02
CMC 3	0.786	0.972	0.985	0.99
ALG-CMC 3	0.881	0.972	0.979	0.97

### Biocompatibility Testing

**Figure 7:**
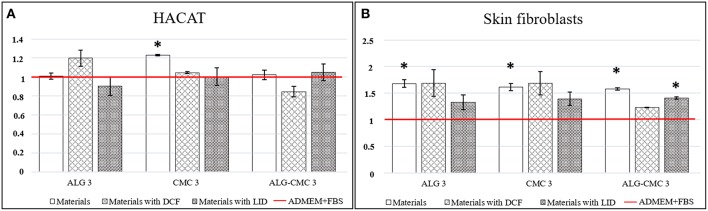
**(A)** HaCaT cells' viability determination using the MTT assay for all samples and the control; ADMEM + FBS (red line in graph), **(B)** Skin Fibroblast cells' viability determination using the MTT assay for all samples and the control; ADMEM + FBS (red line in graph). Error bars represent standard deviation of the replicate measurements. Statistical significance is defined as ^*^P < 0.05 (ANOVA test) for all samples compared to the control.

*In vitro* biocompatibility testing with human skin cells is an important indication for developed material safety and (depending on the type of material and purpose) to some extent efficiency for wound application. In comparison to most available studies, where animal-derived or cancer-transformed cells are used, the results obtained using human cells can serve as a much better approximation of the actual influence of the developed materials in clinical use (Stana et al., 2017). The viability of keratinocytes (HaCaT) and skin fibroblasts (SF) with the drug incorporated multi-layers was tested to confirm the potential of prepared materials for wound care applications. Since extracts of the developed materials were tested against the skin cells, pure cell growth media (ADMEM + FBS) was used as the control sample. From Figure 7, which show the obtained results of this testing, it is immediately apparent that all samples with one exception among each of the developed drug filled multi-layer systems, outperformed the control sample. The exception is LID incorporated ALG3 and DCF incorporated ALG-CMC3 multi-layer; however, no significant deviation was measured even in these samples, as well as no differences in the cell morphology was detected for neither of these samples, indicating that no toxicity whatsoever is present. Important conclusions, which can be made here; no toxic degradation products are formed in neither of the samples (namely, these would significantly affect the cell growth in a negative sense), and the tested multi-layers promoted the growth of cells, especially the skin fibroblasts (Figure 7B). After 48 h, the viability of skin fibroblasts significantly increased in almost all cases, whereas the best results were obtained for the drug-free and DCF included ALG3 multi-layer. Unexpectedly, the incorporation of LID did partially lower the viability of the cells compared to the drug free materials, although the values still remained above the control. Similar results were acquired for skin fibroblasts exposed to CMC 3 with and without included drugs. In the case of ALG-CMC 3, the viability decreased a little after addition of DCF, but nevertheless it remained above the one measured for the control sample. Similar results, only with somewhat lower values, were obtained in the case of HaCaT cells.

Altogether, the obtained results prove that regardless of the polymer-drug combinations used in this study, especially when considering the different mechanisms of action of the LID and DCF drugs, they did not exhibit any toxic effect on neither of the most abundant human skin cells (keratinocytes and fibroblasts). Therefore, we can conclude that tested materials with and without included drugs are biocompatible. The measured higher viabilities in most sample cases compared to the control, require further studies to evaluate the physiological mechanism behind, whereas at the moment, the obtained results indicate that the developed materials are very suitable for use in wound care.

## Conclusions

A simple and rapid method to fabricate nanoscale multi-layered system from water soluble polysaccharides using the combination of spin-coated and LBL techniques is demonstrated. The potential application of multi-layered thin films created from ALG and CMC for the effective incorporation and controlled release of two analgesic drug molecules (DIC and LID) at a physiological condition is investigated in detail. Upon spin-coating, the phase separation occurred for the ALG-CMC mixture, which could not be seen for either ALG or CMC. Such phase separated features are not influenced by the incorporation of either DIC or LID. The presence of latter can be confirmed by ATR-FTIR measurements; however, no substantial changes in the surface wettability are observed before and after the incorporation of drug molecules. Although the film thickness is increased for all three system with a number of deposition steps, a thicker film (highly adsorbed mass) is formed for both CMC and ALG-CMC. A rapid “burst” release followed by a prolonged release kinetics are observed for both drugs regardless of the multilayered system, while the overall released mass is lower for ALG-CMC compared to either ALG or CMC coated surfaces alone. The release properties of both drugs are suited for Korsmeyer–Peppas model, which showed a best fit and the highest “*r*^2^ value” when compared to other two models. The release behavior is predicted to be non-Fickian based on the exponent “n” value which is above 0.89 in all cases. Although the influence of incorporated drugs in the multi-layered thin films is not significant in the growth of both tested skin cells, a pounced growth of skin fibroblast cells is detected already for drug-free multi-layered films. Overall, the knowledge gained through this study is highly beneficial not only to develop a highly multifunctional materials but also for applications related to targeted drug-delivery and tissue engineering, in general.

## Data Availability

All datasets generated for this study are included in the manuscript and/or the Supplementary Files.

## Author Contributions

TiM performed all experiments and contributed substantially to the writing of this manuscript. TaM contributed substantially to the writing, handled submission and revising of the manuscript. LG performed experimental work related to cell testing. MF, KS, and UM contributed to writing and revising of the manuscript.

### Conflict of Interest Statement

The authors declare that the research was conducted in the absence of any commercial or financial relationships that could be construed as a potential conflict of interest.
