# Supplementary material for: Polysaccharide Thin Solid Films for Analgesic Drug Delivery and Growth of Human Skin Cells
Source: Front Chem. 2019 Apr 9;7:217. doi: 10.3389/fchem.2019.00217 (PMC6466929; doi:10.3389/fchem.2019.00217)

Supplementary Material

**Polysaccharide Thin Solid films for Analgesic Drug Delivery and Growth of Human Skin Cells**

Tina Maver^1,2^*, Tamilselvan Mohan^1^*, Lidija Gradišnik^3^, Matjaž Finšgar^4^, Karin Stana Kleinschek^1,5^, Uroš Maver^2,3^

^1^Laboratory for characterization and processing of polymers, Faculty of Mechanical Engineering, University of Maribor, 2000 Maribor, Slovenia

^2^Department of Pharmacology, Faculty of Medicine, University of Maribor, Maribor, Slovenia

^3^Institute of Biomedical Sciences, Faculty of Medicine, University of Maribor, Maribor, Slovenia

^4^Faculty of Chemistry and Chemical Engineering, University of Maribor, 2000 Maribor,

Slovenia

^5^Institute for Chemistry and Technology of Materials, Graz University of Technology, Stremayrgasse 9, 8010 Graz, Austria

*** Correspondence:**Corresponding Authors
[tina.maver@um.si](mailto:tina.maver@um.si); [tamilselvan.mohan@um.si](mailto:tamilselvan.mohan@um.si)


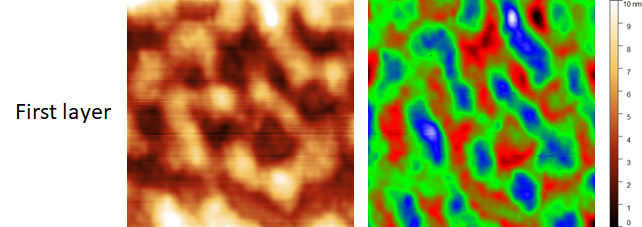


**Figure S1**. AFM height image (1 x 1 μm^2^) of the first ALG-CMC spin coated layer. Both (left and right) images are the same but are presented in different colors to confirm the existence of the phase separation.


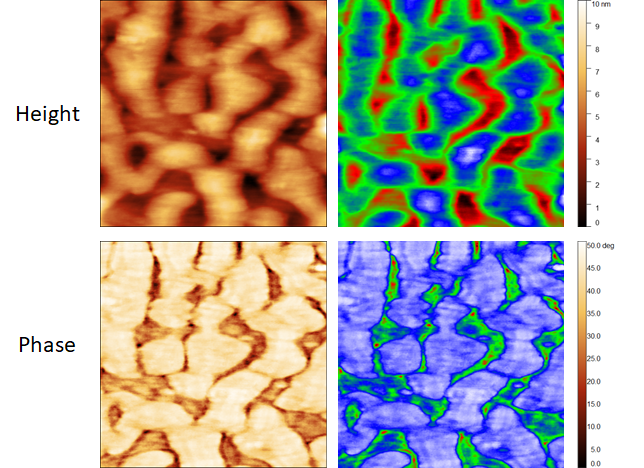


**Figure S2**. AFM height (top) and phase (bottom) image (1 x 1 μm^2^) of the third ALG-CMC spin coated layer. Both (left and right) images are the same but are presented in different colors to confirm the existence of the phase separation. In the phase image, the presence of two phases can be clearly seen.

**Table S1.** Static contact angle (SCA) values of alginate (ALG), carboxymethyl cellulose (CMC) and ALG-CMC spin coated multi-layers, incorporated with and without drugs.


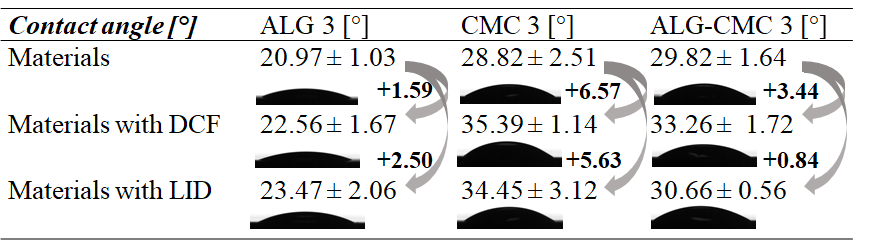

Supplement: Supplementary file 1 [file Data_Sheet_1.docx]
